# Handheld NIR Spectral Sensor Module Based on a Fully-Integrated Detector Array

**DOI:** 10.3390/s22187027

**Published:** 2022-09-16

**Authors:** Fang Ou, Anne van Klinken, Petar Ševo, Maurangelo Petruzzella, Chenhui Li, Don M. J. van Elst, Kaylee D. Hakkel, Francesco Pagliano, Rene P. J. van Veldhoven, Andrea Fiore

**Affiliations:** 1 Department of Applied Physics and Eindhoven Hendrik Casimir Institute, Eindhoven University of Technology, P.O. Box 513NL, 5600 MB Eindhoven, The Netherlands; 2 MantiSpectra B.V., De Groene Loper 3, 5612 AE Eindhoven, The Netherlands

**Keywords:** spectral sensing, near-infrared, sensors, portable devices, integrated photonics

## Abstract

For decades, near-infrared (NIR) spectroscopy has been a valuable tool for material analysis in a variety of applications, ranging from industrial process monitoring to quality assessment. Traditional spectrometers are typically bulky, fragile and expensive, which makes them unsuitable for portable and in-field use. Thus, there is a growing interest for miniaturized, robust and low-cost NIR sensors. In this study, we demonstrate a handheld NIR spectral sensor module, based on a fully-integrated multipixel detector array, sensitive in the 850–1700 nm wavelength range. Differently from a spectrometer, the spectral sensor measures a limited number of NIR spectral bands. The capabilities of the spectral sensor module were evaluated alongside a commercially available portable spectrometer for two application cases: to quantify the moisture content in rice grains and to classify plastic types. Both devices achieved the two sensing tasks with comparable performance. Moisture quantification was achieved with a root mean square error (RMSE) prediction of 1.4% and 1.1% by the spectral sensor and spectrometer, respectively. Classification of the plastic type was achieved with a prediction accuracy on unknown samples of 100% and 96.4% by the spectral sensor and spectrometer, respectively. The results from this study are promising and demonstrate the potential for the compact NIR modules to be used in a variety of NIR sensing applications.

## 1. Introduction

Near-infrared spectroscopy (NIRS) is a method that has been used for decades to study the properties of materials from their reflection or transmission spectrum in the 800–2500 nm wavelength range [1]. NIRS is widely used today in applications ranging from monitoring industrial manufacturing processes to assessing the chemical composition and quality of products and materials. The advantages of NIRS include that it is fast, has a non-destructive nature, requires minimal sample preparation and no chemical consumables and has the ability to provide information on multiple constituents from the same measurement data. However, typical benchtop NIR spectrometers are large, expensive, complex and include moving parts, making them sensitive to vibrations and shocks.

The miniaturization of spectrometers is essential to expand their application beyond dedicated stations in industrial settings and analytical labs, into the hands of non-specialists working on-site and eventually to consumers. Technological advances in photonics and fabrication have enabled cost and size reduction leading to on-site and portable NIR spectrometers, with different wavelength ranges and spectral resolutions [2,3].

The design of portable NIR spectrometers is mostly inspired by conventional benchtop instruments that use gratings or interferometers. Common existing portable spectrometers are based on compact gratings with single detectors or detector arrays, in some cases combined with microelectromechanical structures (MEMS) [4,5,6,7,8,9], linear-variable filters coupled to detector arrays [10,11] and interferometers combined with MEMS [12,13]. NIR wavelengths above about 1050 nm fall outside of the silicon detection region and other materials, such as InGaAs, are required for detection, leading to a high cost for approaches using large InGaAs arrays. For this reason, single-pixel approaches with an InGaAs detector, using, e.g., a digital light processor (DLP), have been pursued in NIRS [5]. This approach is suitable for handheld devices but further miniaturization, e.g., for integration into a mobile phone, is limited by the size of the DLP. In all these cases, the detector is not integrated, resulting in optical loss and complex packaging. Furthermore, MEMS approaches contain movable parts that are sensitive to shocks and mechanical vibrations. Another avenue pursued for spectrometry is the use of photonic integrated circuits, where detector integration is feasible [14,15]. However, the spectral range of such circuits is limited, and they require a single-mode input, which is incompatible with most applications which use diffuse transmittance or reflectance, and therefore involve spatially incoherent light.

Recently, a novel approach to NIR spectral sensing was proposed, using a miniaturized fully-integrated multipixel array of resonant-cavity-enhanced (RCE) InGaAs photodetectors [16]. This is different than conventional spectrometry in the sense that the sensor does not exactly measure the spectrum but rather a limited number of spectral regions, with a limited resolution (50–100 nm). The sensor chip has a footprint of 1.8 × 2.2 mm^2^ and consists of an array of 16 pixels with tailored spectral responses in the 850–1700 nm wavelength range. Each pixel is fabricated within a single monolithic element having a thin absorbing layer and a tuning layer inside an optical cavity. In this approach, the detector and filter elements are directly co-integrated at the wafer level, providing a robust system which can be fabricated at high volumes using standard semiconductor processing methods [16]. The compatibility for volume production is key as this enables the cost to scale down as demand increases.

**Figure 1:**
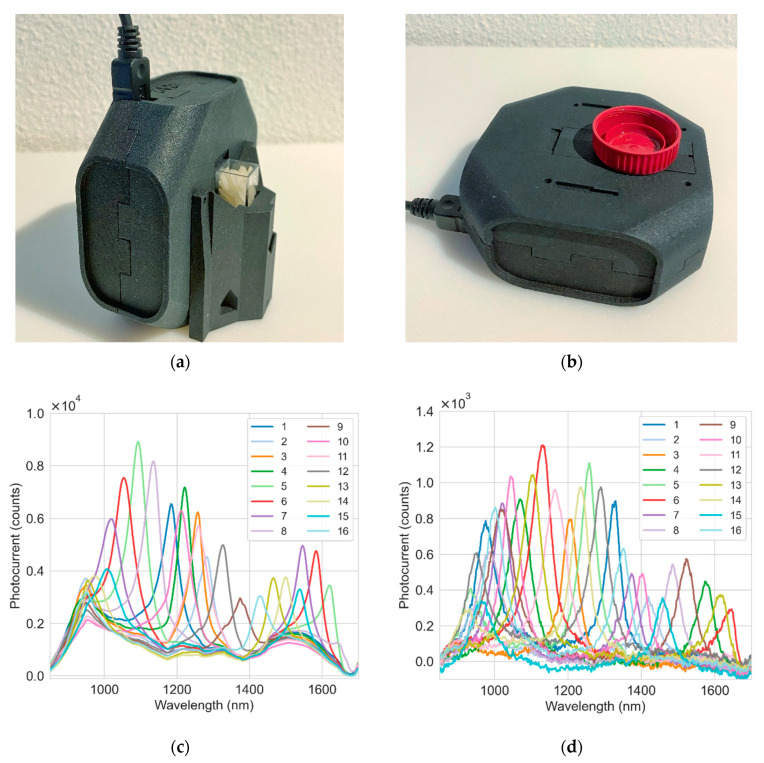
The standalone handheld SpectraPod modules (physical dimension: 8.2 × 8.2 × 3.4 cm^3^) and their response curves. (**a**) SpectraPod with cuvette holder attachment used in the moisture quantification experiment; (**b**) the basic SpectraPod module for acquiring reflectance measurements from plastic samples. The response curves of the SpectraPod containing chip 1 (**c**) and chip 2 (**d**).

In this study, we describe a standalone handheld sensing module (Figure 1a,b), referred to as SpectraPod^TM^ (MantiSpectra, Eindhoven, The Netherlands), based on this new NIR sensing concept. The module incorporates the 16 pixel integrated chip along with an internal light source and basic optics for light collection. The performance of the SpectraPod module was evaluated and compared to a commercial miniature NIR spectrometer, the NIRScan^TM^ (Texas Instruments Inc., Dallas, TX, USA) in two exemplary real-world application cases: a quantification of the moisture content (MC) in rice grains and plastic type classification. The NIRscan is an example of the MEMS-based DLP approach. It incorporates a MEMS digital micromirror device, a diffraction grating and a single point detector [9,17], and features a resolution of ~10 nm in a similar wavelength range (900–1700 nm) as the SpectraPod. Signal detection in the NIRscan is achieved by a 1 mm diameter InGaAs detector (Hamamatsu Photonics, Hamamatsu, Japan; model G12180-010A). The purpose of this comparison is to assess whether an integrated spectral sensor with a very limited resolution and number of spectral points can deliver a performance comparable to a compact spectrometer in practical application cases.

Moisture quantification in rice and plastic type classification were chosen as representative of a wider class of sensing problems that fall into either the regression or classification category. Furthermore, rice is one of the most importable staple foods in the world, and its level of moisture not only affects its starch, fat and protein content, but is also a crucial parameter to monitor for the drying, storage and refinement processes [18,19]. On the other hand, plastic is one of the most widely used materials and the improvement of its sorting and recycling processes is crucial to reduce the enormous amount of plastic waste generated globally [20,21,22]. NIR spectral sensing has the potential to make a positive impact in both of these important application cases and the miniaturization of the sensor will facilitate access to and adoption of the technology.

## 2. Materials and Methods

### 2.1. The Handheld SpectraPod Module

The NIR diffuse reflectance from both the moisture quantification and plastic classification experiments was measured using a novel, standalone handheld spectral sensing module called the SpectraPod. The core technology of the SpectraPod is the ChipSense^TM^ (MantiSpectra, Eindhoven, The Netherlands) sensor chip, an array of 16 detectors which have tailored broadband spectral responses and exhibit a high peak responsivity (>0.25 A/W) and low noise, enabling measurements at the few pW level. The detector device structure has been described elsewhere [16,23].

The detector array is incorporated into the SpectraPod module along with an adjustable internal miniaturized tungsten lamp, a diffuser, a 850 nm high-pass filter and a lens to focus the incoming light onto the sensor array. The 16 pixels are read out sequentially and the integration time of each pixel ranges from 0.3 to 145 ms (the corresponding read-out time of the whole array is about 16 times longer). Signals from the sensor array are first amplified, then multiplexed and digitized via a 24-bit analog-to-digital converter (ADC). The ADC is interrogated using a microprocessor which relays the data to a computer via a USB connection. Various illumination and signal collection configurations are available via the attachment of several extensions, including reflectance, interactance, transmission and a fiber-coupled input. Rice samples for the moisture quantification experiment were placed in cuvettes and measured in reflectance mode using a cuvette holder extension (Figure 1a). The samples for the plastic classification experiment were measured directly in the basic reflectance configuration (Figure 1b). The SpectraPod was operated via SpectraByte^TM^ (v2.1, MantiSpectra, Eindhoven, The Netherlands), an in-house developed software application. The application controls the instrument and acquisition settings, including the integration time, number of scan averages, lamp power and operation mode.

Two SpectraPods were used in the two experiments, and each contained a different version of the ChipSense sensor chip. The moisture quantification experiment used a first generation of chips (“chip 1”), whereas a second generation (“chip 2”) was used for the plastic classification experiment. The response curves of the two sensor chips are shown in Figure 1c,d, which were measured as a function of the illumination wavelength using a narrow spectral line produced by a monochromator (8 nm linewidth, about 1.34 μW power). Both chips had pixel linewidths varying from 40 to 90 nm. Chip 1 had a higher peak responsivity but also had a significant non-resonant background, which is suppressed in chip 2 by reducing the absorption outside of the resonant cavity [23]. Chip 2 also had lower responsivity. Due to the electrical connections, the pixels of chip 2 may be slightly biased, leading to measurements with a negative photocurrent (Figure 1d).

### 2.2. Sample Preparation for Rice Moisture Quantification Experiment

Two types of grains, the short-grain Arborio rice (Riso Scotti, Pavia, Italy) and long-grain Jasmine rice (Tilda, Rainham, England) were used in the experiment. The two types of rice grains remained separate from each other and both types were subject to the same sample preparation and measurement protocols in parallel. The wet basis MC is used in this paper, and it is calculated using the weight loss due to water evaporation during the oven-drying process (based on ISO 712:2009) and taking the ratio of this change with the original sample weight.

The rice grains were removed from their packaging and directly soaked overnight for about 9 h at room temperature. Then, the soaked grains were placed in a thin layer onto a dry towel for about 2 h to allow for the evaporation of superficial water. After this step, the oven-drying method was used to measure the initial MC of these moist grains. These moist grains were weighed and then further dried inside an oven set to 130 °C for 120 min. Following the initial 120 min of drying, the process was repeated for another 60 min of drying to ensure no further changes in weight. The final weight of the grains after this extensive drying process was measured and the initial MC of the moist grains was calculated.

Two batches of calibration samples for each grain type were prepared using the moist grains that were superficially dried after overnight soaking. Each batch was dried separately in an oven at 50 °C with drying times of 5, 15, 25, 40, 70, 115, 160 and 220 min to obtain calibration samples with varying MCs. After each drying interval, each batch was cooled to room temperature and weighed for MC determination, and two subsamples of the grains from each batch were transferred into two cuvettes for optical measurement. One batch of independent test samples for each grain type was prepared in a similar way to the calibration samples, using the superficially dried grains. Test samples with varying moisture content were obtained via oven-drying at 50 °C with durations of 20, 40, 60 and 120 min.

### 2.3. Sample Preparation for Plastic Classification Experiment

**Figure A1:**
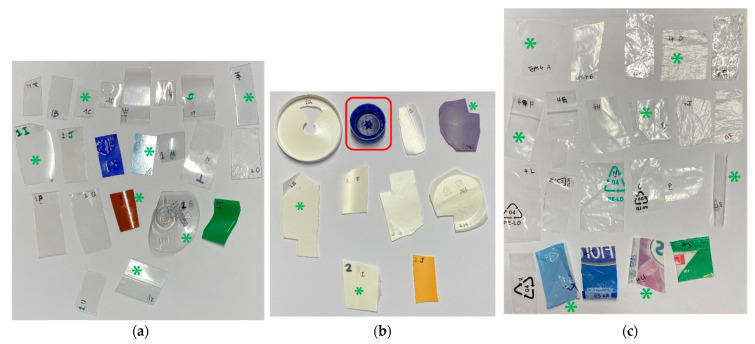

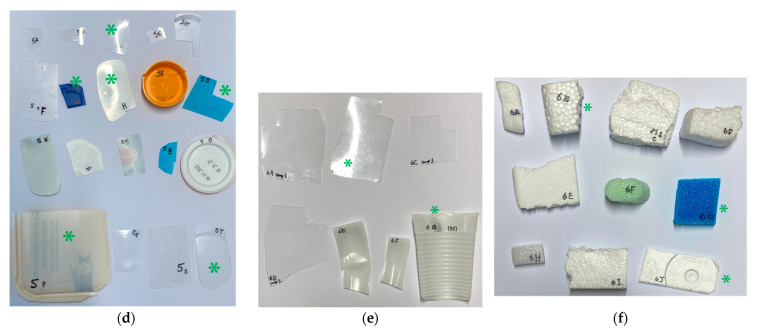
Photograph showing the appearance of samples measured for the plastic classification experiment. (**a**) type 1 PET; (**b**) type 2 HDPE (dark blue sample that resulted in low reflectance is circled in red); (**c**) type 4 LDPE; (**d**) type 5 PP; (**e**) type 6 PS; and (**f**) type 6 foam. The green stars indicate the samples that were used in the test set.

Ninety plastic samples with various thicknesses, colors and textures were used in this study, which were collected by members of the research group from their household waste. Twenty-eight of the 90 samples were selected in a randomized way prior to measurement as the test set, such that this group consisted of 7× type 1 (polyethylene terephthalate; PET), 3× type 2 (high-density polyethylene; HDPE), 7× type 4 (low-density polyethylene; LDPE), 6× type 5 (polypropylene; PP), 2× type 6 (polystyrene; PS) and 3× type 6 foam (PS foam). All the remaining 62 samples were incorporated in the training set. An overview of the training and test sample sets and their appearances can be found in Table A1 and Figure A1 in Appendix A.

### 2.4. Spectral Data Acquisition

The samples were measured using two SpectraPods, with chip 1 for moisture quantification and with chip 2 for plastic classification. The samples were illuminated by a single 45° angled internal tungsten lamp. Each measurement obtains 16 photocurrent values from the array of 16 pixels which were directly used in chemometrics analysis without the need for spectral reconstruction.

In addition to the SpectraPod, samples from both experiments were also measured by the handheld spectrometer NIRscan. The NIRscan illuminates using two 45° angled internal tungsten halogen lamps [24,25]. Each measurement obtains up to 566 measurement points, corresponding to discrete wavelengths in the 900–1700 nm range.

#### 2.4.1. Moisture Quantification Experiment

For the moisture quantification experiment, the samples for optical measurements were placed in a cuvette then measured by the SpectraPod in 3 acquisition cycles (145 ms integration time per pixel, about 10 s total per measurement) and by the NIRscan in 32 acquisition cycles (0.64 ms integration time per point with 330 measurement points, about 15 s total per measurement). The cuvettes containing rice samples were placed against the respective measurement window of the SpectraPod and NIRscan for measurement. The data from the different acquisition cycles were averaged. Each cuvette of rice sample was measured two times. The rice samples were measured by the NIRscan immediately after measurement by the SpectraPod and the same number of spectral measurements was obtained by both devices. The lamp power used in the experiments with the SpectraPod was about 7 times lower than the one used with the NIRscan.

#### 2.4.2. Plastic Classification Experiment

Samples for the plastic classification experiment were measured sequentially by both the SpectraPod and NIRscan by directly placing the samples against their respective measurement windows. Four different locations were measured per sample. Translucent and transparent plastic samples were measured with a diffuse reflectance standard (>95% reflectivity; Ocean Insight, Duiven, The Netherlands) held against the sample. One acquisition cycle was used for both the SpectraPod (145 ms integration time per pixel) and NIRscan (5.08 ms integration time per point with 566 measurement points), the total time per measurement was about 3 s for both devices. The lamp power used with the SpectraPod was in this case about 3.5 times lower than the one used with the NIRscan.

### 2.5. Data Analysis

#### 2.5.1. Moisture Quantification

The 16 photocurrent values obtained by the SpectraPod were dark-corrected via subtraction of the photocurrent values acquired under the darkness. Outlier analysis was done using a partial least squares (PLS) model to obtain the Q^2^ residuals and Hotelling’s T^2^, which indicates the variation remaining in each sample after projection through the model and the distance between each sample and the multivariate mean within the model, respectively [26]. Measurements with abnormally high variance from the expected means and not belonging to samples containing the highest or lowest MC were identified as outliers. Three out of 176 measurements of the entire sample set were identified as outliers and excluded from subsequent analysis.

After excluding the outliers, the dark-corrected photocurrent values from the repeated measurement on each cuvette were averaged (i.e., each cuvette was measured twice which resulted in one averaged measurement per sample). Subsequently, two preprocessing methods were applied and compared: mean-centering and sum normalization (normalization by the sum of all the spectral data points in each measurement).

PLS regression (PLSR) was used to model MC in rice grains using the calibration sample set and cross-validation (CV) was used to optimize the number of latent variables (LVs) used in the model. Five-fold CV was used whereby in five iterations, a different 20% of the calibration data was held out as the internal validation set while the remaining were used to train the model. The variations of the root mean square error of calibration (RMSEC) and cross-validation (RMSECV) were evaluated for increasing numbers of LVs. The subset of LVs, whose contributions appreciably decreased the RMSECV without making the RMSECV diverge strongly from the RMSEC, was retained and the resulting optimized model was used for subsequent evaluation of the test sample set. No distinction was made between measurements from the two grain types (Arborio/Jasmine), i.e., information on the grain type was not used in the model.

The equivalent data processing and modelling procedures described above were also applied to the spectral data measured by the NIRscan. However, in addition to mean-centering and sum normalization, standard normal variate, Savitzy–Golay with first derivative and Savitzy–Golay with second derivative, were also compared for preprocessing of spectral data obtained by the NIRscan and the approach providing the best cross-validation performance was chosen. These additional preprocessing methods were not applied to SpectraPod data due to those having only 16 datapoints per measurement and that the datapoints were not correlated with each other. The NIRscan measurements for MC quantification consisted of 330 spectral data points (digital resolution), spanning 900–1700 nm in about 2.4 nm steps. Two out of the total 176 NIRscan measurements were identified as outliers and excluded from subsequent analysis.

In all cases, the statistical measures used to assess the prediction performance of the models included the coefficient of determination (R^2^) and the ratio of prediction error to standard deviation (RPD). The errors were quantified using RMSEC and RMSECV which were calculated using the calibration data set and the RMSE of prediction (RMSEP) calculated using the test set.

#### 2.5.2. Plastic Classification

The photocurrent values from each SpectraPod measurement were dark corrected and then outliers were identified by plotting their Q residuals and Hotelling’s T^2^ (obtained from PLS decomposition). Four out of the total 360 measurements were identified as outliers and excluded. Then, the four replicate measurements on different locations of each plastic sample were combined into one averaged measurement per sample. Subsequently, two preprocessing methods were again compared: mean-centering and sum normalization.

Classification models were built using the calibration sample set and six methods were compared: principal components analysis-linear discriminant analysis (PCA-LDA), partial least squares-discriminant analysis (PLS-DA), support vector machine (SVM), random forest (RF) and PLS-RF. In all cases, 5-fold CV was used to optimize the classification models. The combination of the preprocessing method and classifier that resulted with the highest accuracy in CV was used in the evaluation of the test samples and is shown in this manuscript.

Equivalent data processing and modelling procedures were also applied to the spectral data measured by the NIRscan. Additional preprocessing using the standard normal variate, Savitzy–Golay with first derivative and Savitzy–Golay with the second derivative, were also compared for the NIRscan data, and the approach providing the best cross-validation performance was chosen. The NIRscan measurements for plastic classification consisted of 566 spectral data points, spanning 900–1700 nm in about 1.4 nm steps. Five out of 360 NIRscan measurements were identified as outliers and excluded from subsequent analysis.

All data analysis algorithms were implemented in Python (Python Software Foundation, Beaverton, OR, USA) using packages from NumPy [27], Matplotlib [28] and Scikit-learn [29].

## 3. Results

### 3.1. Moisture Quantification Experiment

#### 3.1.1. Spectral Measurements

**Figure 2:**
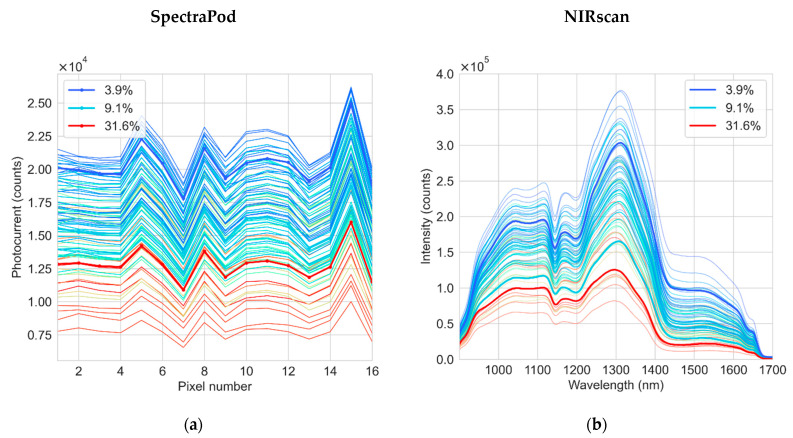

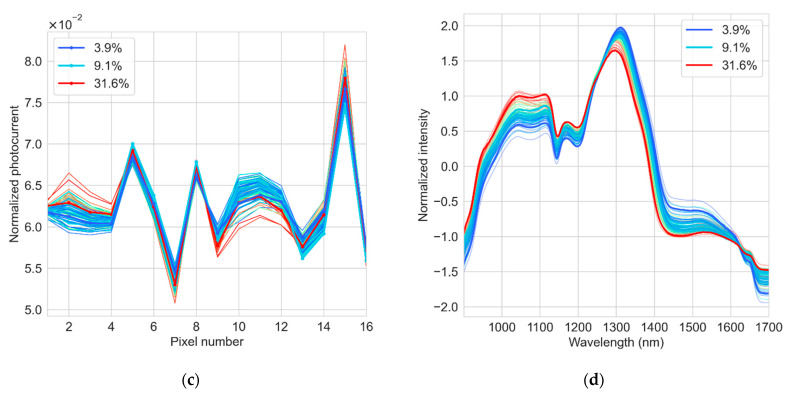
The raw photocurrent and intensity measurements collected using the (**a**) SpectraPod and (**b**) NIRscan, (**c**) the transformation of SpectraPod measurements after normalization by the sum of all spectral points within each measurement and (**d**) the transformation of NIRscan measurements after applying the standard normal variate method. One measurement corresponding to the minimum, median and maximum moisture content is highlighted by the bold line in each plot and their corresponding MC is indicated in the legend.

The wet basis MC of the rice grain samples obtained using various oven-drying time intervals ranged from 3.9 to 31.6%. Figure 2a,b shows the raw signals measured from both the SpectraPod and the NIRscan. In both devices, there is a general increase in the measured signal strength as the MC of the samples decreased. The water absorption bands are located at 970, 1190 and 1450 nm [30], producing broad features as shown in Figure 2b. The variation in spectral shape in relation to the change in MC becomes more apparent following the normalization of the SpectraPod or NIRscan measurements (Figure 2c,d). The normalization method that resulted in the PLS regression model with the lowest RMSECV was sum normalization for the SpectraPod data and standard normal variate for the NIRscan data.

#### 3.1.2. Quantification of Moisture Content in Rice Grains

*N* = 72 calibration samples (*n* = 144 spectral measurements) and *N* = 16 test sample measurements (*n* = 32 spectral measurements) were measured by both the SpectraPod and NIRscan. PLS was used to build a regression model for each device to quantify the MC in the two types of rice grains used in the experiment. The sum normalized photocurrent values from the SpectraPod measurements, which consists of only 16 values, were used directly as input to building the PLSR model without further need for spectral reconstruction. In comparison, each NIRscan measurement consists of 330 intensity values which were normalized using the standard normal variate method before being used in model building.

**Figure 3:**
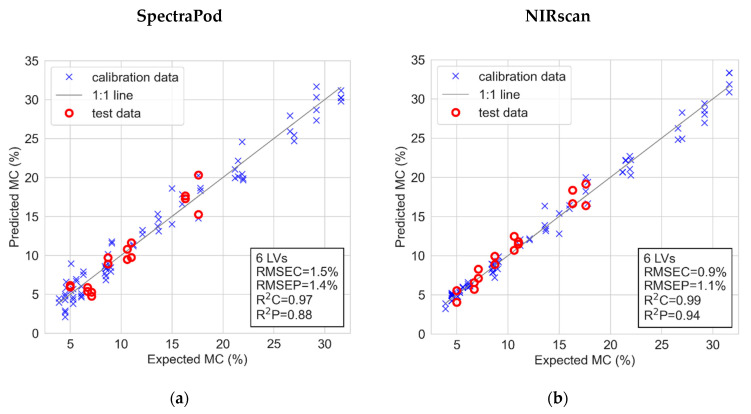
The predicted moisture content of two types of rice grains, obtained via PLS regression using normalized photocurrents from the SpectraPod (**a**) and NIRscan (**b**), compared to their expected values, obtained via the oven-drying method. The number of latent variables (LVs), the root mean square error of calibration (RMSEC) and prediction (RMSEP) and the coefficient of determination for the calibration (R^2^C) and prediction (R^2^P) are indicated.

Figure 3a,b shows the modelling and prediction of MC using the SpectraPod and NIRscan measurements, respectively. Each model was developed using calibration samples and was subsequently evaluated using the corresponding test samples for external validation (model statistics in Table A2). The PLSR model of the NIRscan used six LVs decomposed from an original data structure containing 330 variables (wavelengths), whereas the model of the SpectraPod used six LVs decomposed from a much reduced data structure containing 16 variables (pixels). Nonetheless, the SpectraPod and NIRscan obtained comparable model performance, with the NIRscan achieving a slightly higher accuracy with an R^2^C of 0.97 and 0.99, respectively, and an RMSEP of 1.4% and 1.1% MC, respectively. Both devices obtained high RPD values, 5.0 and 7.3 for the SpectraPod and NIRscan, respectively.

### 3.2. Plastic Classification Experiment

#### 3.2.1. Spectral Measurements

**Figure 4:**
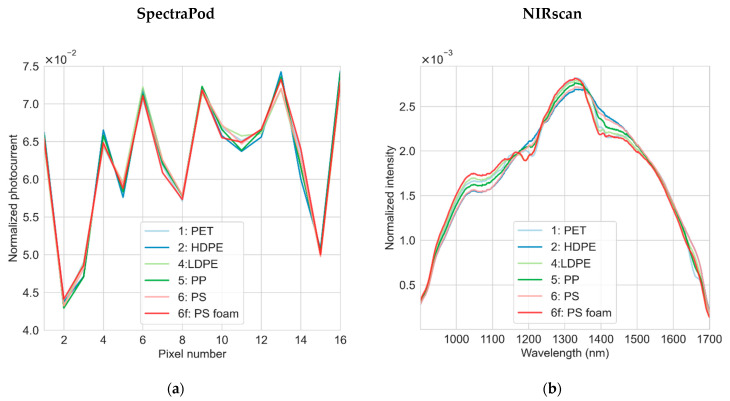
The average measurement of each plastic type measured by the SpectraPod (**a**) and NIRscan (**b**), following normalization by the sum of all spectral points within each measurement.

**Figure A2:**
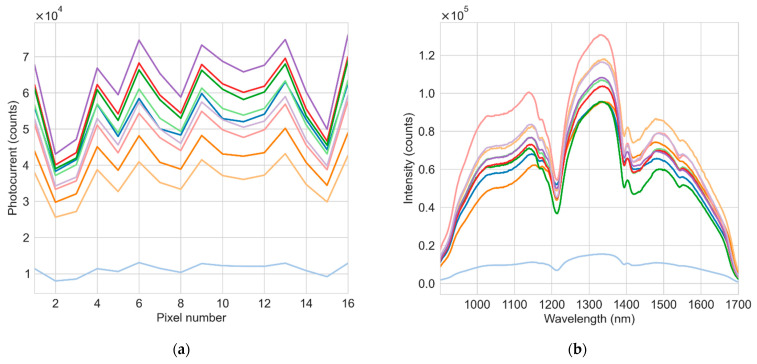
Representative measurements prior to normalization, of colored HDPE (type 2) samples taken by the SpectraPod (**a**) and NIRscan (**b**). The measurement with the lowest photocurrent and intensity resulted from the dark blue sample (circled in red in Figure A1b).

Each plastic sample was measured at four different positions by both the SpectraPod and NIRscan. *N* = 62 calibration samples (*n* = 248 spectral measurements) and *N* = 28 test samples (*n* = 112 spectral measurements) were measured by each device. The average NIR reflectance spectra of each type of plastic measured by the NIRscan, normalized to the sum of the spectral points, is displayed in Figure 4b, showing distinct differences in the spectral shape. Within each plastic type, the overall form of the spectra was maintained, although there were variations in the amplitude of the measured signals (Figure A2) which can be mostly ascribed to variations in the sample thickness and color [20,21]. The spectral differences between the different plastic types are also seen in the SpectraPod measurements, as shown by the difference in the shape of the normalized photocurrent peaks (Figure 4a).

#### 3.2.2. Classification of Plastic Types

**Figure 5:**
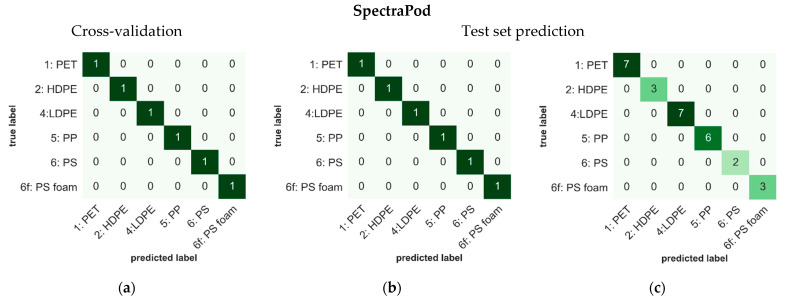

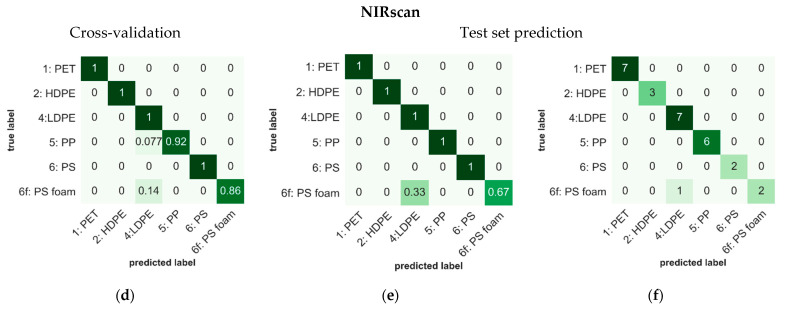
Results of plastic type classification using the SpectraPod (top, **a**–**c**) and NIRscan (bottom, **d**–**f**) measurements. Confusion matrices display the outcome of PLS-DA classification on the calibration (left: relative frequency) and test sample set (middle: relative frequency, right: absolute frequency). One hundred percent accuracy was obtained by the SpectraPod for both cross-validation and test set prediction. Accuracies of 96.9% and 96.4% were obtained by the NIRscan for cross-validation and test set prediction, respectively.

The classification of different plastic types was achieved using spectral measurements of the SpectraPod and NIRscan which contained 16 and 566 variables (pixels or wavelengths) per measurement, respectively. The classification models for both devices were developed using PLS-DA, with sum-normalized spectral data as input. For the SpectraPod, the classification model was developed using 10 PLS latent variables and achieved 100% accuracy in both cross-validation and test set prediction (confusion matrices in Figure 5a–c). In comparison, the classification model of the NIRscan was constructed using six PLS latent variables and achieved an overall accuracy of 96.9% for cross-validation and 96.4% in test set validation (confusion matrices in Figure 5d–f).

## 4. Discussion

### 4.1. Moisture Quantification Experiment

The PLS regression models were built using spectral data from the SpectraPod and NIRscan to predict the MC in Arborio and Jasmine rice in a single model. As shown above, a low RMSEP of 1.4% and 1.1%, and high RPD values of 5.0 and 7.3, were obtained by the SpectraPod and NIRscan, respectively. The RPD value considers both the standard deviation of the reference and error of the prediction, and, generally, an RPD value below 1.5 indicates poor prediction performance, whereas an RPD above three is accepted as an acceptable working model [31,32]. Both devices performed well in this moisture quantification experiment, even though measurements from two very different rice types (short-grain Arborio and long-grain Jasmine) were combined in the modelling process. The results suggest that despite differences in the breeds (and in turn differences in shape, outer shell thickness and chemical compositions) it is feasible to use one regression model based on the NIR measurements for monitoring the MC in both rice types. This observation should be further tested on other types of rice, e.g., brown and purple rice, and validated over a larger MC range.

Our results compare well with previous studies that have applied NIR spectroscopy to the quantification of MC in rice grains. For example, Makky et al. measured two local rice cultivars of Indonesia, using an FT-NIR spectrometer (1000–2500 nm) [19]. The two cultivars were modelled separately in this study and each model obtained a RPD value of about 3.9 and 1.6 (as calculated from the reported data). Lin et al. measured the MC of only one type of long-grain rice using a portable NIR spectrometer (950–1650 nm), and a RPD value of 6.01 (as calculated from the reported data) was obtained [18].

### 4.2. Plastic Classification Experiment

Classification models for the discrimination of different plastic types was built for both the SpectraPod and NIRscan, using PLS-DA. Both devices performed well, obtaining test set validation accuracies of 100% and 96.4% by the SpectraPod and NIRscan, respectively. Two misclassifications occurred in the cross-validation analysis of the spectrometer data and one misclassification occurred in the prediction of unknown test samples. Overall, these misclassifications are minor compared to the majority of correct classifications. While it may be possible to also obtain 100% accuracy from the spectrometer data using other analysis methods, in the current study we chose to keep the data analysis of the two devices comparable. The distinct reflectance signals measured in the NIR range by the two devices (850–1700 nm for the SpectraPod and 900–1700 nm for the NIRscan) is mostly due to the overtone vibrations of the plastic molecules. C-H, O-H, N-H and C-O bonds have distinct absorption peaks in this NIR region, which give rise to the spectral characteristics of the plastic samples and enables the identification of their type [20,21,33].

The overall spectral characteristics in the NIR are maintained despite differences in sample thickness and variations in color [20]. The spectral measurements resulting from variations in color, thickness and surface roughness are exemplified in Figure A2, where measurements of the HDPE (type 2) samples are shown. Even one sample with a dark blue color, resulting in an average reflectance about a factor of five smaller than other samples in the group, could still be classified correctly (in cross-validation). However, this may not work for black-colored samples. Black samples have a very low reflectance in the NIR spectral region which hampers their classification using NIR spectroscopy [20,34]. Instead, mid-IR (MIR; 3–12 µm) spectroscopy can be used, as black plastic samples have pronounced MIR spectral features [34].

NIR-based spectrometers have been shown to be very promising for fast and reliable determination of plastic types. Masoumi et al. used a desktop spectrometer (700–2000 nm) to measure five types (PET, HDPE, PP, PS and PVC; polyvinyl chloride) of light colored plastics and demonstrated that the spectral signatures can be used to reliably distinguish between each type [20]. Zheng et al. used a NIR hyperspectral imaging system (1000–2500 nm) to measure six types (PET, PE, PP, PS, PVC and ABS; acrylonitrile butadiene styrene) of non-black plastics and achieved a cross-validation accuracy of 84.9% and 100% accuracy in test set prediction [33]. Rani et al. used a handheld NIR spectrometer (900–1700 nm) to measure five types (PET, PE, PP, PS and PVC) of non-black plastics and obtained 100% accuracy in both cross-validation and test set prediction, with 5 (out of 901) and 4 (out of 386) scans not assigned to any of the considered classes, respectively [21].

In the present study, models were developed using common plastic waste collected in the household of the researchers. Further research may be conducted to expand both the number of samples within the existing class types and the range of plastic types, e.g., to include PVC, ABS, polycarbonate and others. In addition, diffuse reflectance standards for the enhancement of the reflectance signal from translucent and transparent samples will most likely not be available in real application scenarios. For translation to real applications of plastic sorting, different diffusely reflecting surfaces, even with a non-flat reflectance spectrum, can be tested. Overall, the findings of this study align with the existing body of work. Additionally, the high classification performance obtained with the SpectraPod indicates that high spectral resolution is not needed for plastic identification using NIR.

### 4.3. Comparison, Limitations and Future Outlook

Overall, for these two applications, the sensing performance obtained with the SpectraPod is comparable to the one obtained with the NIRscan, which shows the potential of the spectral sensing approach with a simple integrated chip. Particularly, in both cases the number of LVs used in the regression models for the NIRscan data is very close to the one used for the SpectraPod data, despite the vastly larger number of spectral points. This is a clear indication that the small ChipSense array efficiently captures the relevant spectral information even if it does not measure the spectrum. An even more efficient spectral sensing hardware may be obtained by optimizing the responsivity spectra for this specific problem [16].

While we expect this conclusion to be applicable to a broad class of NIR sensing problems, there are cases where measurement of the spectrum is needed. Particularly when exploring a sensing application for the first time, it is useful to see the full spectrum, identify where the important features are, and compare with previous studies. The lack of wavelength-specific information in the SpectraPod means that its measurements have lower interpretability compared to the measurements of the full spectrum. It is possible to reconstruct a spectrum from the 16 datapoints measured by the SpectraPod, for example, by using the method proposed in [35]. However, applying this process requires knowledge of both the response function of the sensor and the expected spectral characteristics of the measured sample, making this complex to undertake in a practical scenario. We also note that spectral reconstruction will not increase the prediction accuracy of models, as no new information is added, so it is not relevant for the data analysis. Furthermore, it is also arguable that the insights available in the NIR region are limited in the first place, due to the broad and overlapping absorption bands in this region [36]. NIR sensing is in this sense different from the sensing in the MIR range, where wavelength resolution may be more important, due to the sharper and more diagnostic spectral signatures present in the MIR.

Additionally, the spectral sensing approach used in the SpectraPod may provide reduced performance if the chemical features are sharp (<50 nm) and are in a wavelength region not well covered by the response peaks of the 16 pixels. For example, gas sensing may be difficult, due to the narrow spectral features typically present in the gas phase [37]. Another limitation to the direct sensing by the SpectraPod is that only a subset of chemometrics processing methods can be meaningfully used, due to the reduced number of data points captured in each measurement and the lack of wavelength resolution. For example, although common preprocessing methods such as derivative transformation can be applied mathematically to SpectraPod data, the transformation would not hold the same traditional meaning, as adjacent pixel numbers do not correspond to adjacent wavelength points. On the other hand, the SpectraPod’s reduced number of data points means a simplified and faster data handling and analysis process. Overall, miniaturized handheld NIR devices such as the SpectraPod represent a practical solution for industrial and consumer sensing applications, rather than a replacement of high-end benchtop spectrometers.

## 5. Conclusions

There are continuous developments in the pursuit of portable, handheld miniaturized devices for on-site usage, due to the limitations of price, size and complexity on traditional benchtop NIR spectrometers. In this article, we demonstrated the successful application of a low-cost handheld NIR sensor module, the SpectraPod, based on an array of resonant-cavity-enhanced photodetectors, to quantify the MC in rice grains and to classify plastic types. The capabilities of the SpectraPod were evaluated alongside a mature, commercially available NIRscan miniature spectrometer, showing comparable prediction accuracy. These two types of devices utilize different approaches to spectral sensing in the NIR range. The NIRscan, like conventional spectrometers, measures the spectrum (i.e., data consists of spectral intensity corresponding to discrete wavelengths). On the other hand, the SpectraPod’s approach is closer to the spectral sensing done by our eyes which allows us to perceive color with only three types of cone cells. The results show the capabilities of the SpectraPod’s approach of spectral sensing, which uses only 16 spectral bands, as compared to the measurement of the full spectrum in traditional spectrometers. It also lays the foundation for future studies that extend the application of multipixel spectral sensing to other meaningful analytical problems.

## Data Availability

The data are available from the corresponding author upon reasonable request.

## Appendix A

**Table A1:** Composition of the calibration and test sets for the plastic classification experiment.

Samples (*N* = 90)	Calibration (*N* = 62)	Test (*N* = 28)
1: PET	15	7
2: HDPE	7	3
4: LDPE	15	7
5: PP	13	6
6: PS	5	2
6f: PS foam	7	3

**Table A2:** Model statistics for the prediction of moisture content.

Measurement Device	No. of Variables (Pixels or Wavelengths)	No. of LVs ^a^	Cross-Validation						Validation
			RMSECV ^b^	R^2^	RPD ^c^	Bias ^d^	Slope ^e^	Offset ^f^	RMSEP ^b^
SpectraPod	16	6	1.8	0.96	5.0	−1.91 × 10^−2^	0.96	0.59	1.4
NIRscan	330	6	1.2	0.98	7.3	−4.42 × 10^−2^	0.98	0.19	1.1

^a^ LV = latent variables selected. ^b^ RMSECV and RMSEP = root mean square error of cross-validation (CV) and prediction (P), expressed in % (wet basis moisture content). ^c^ RPD = ratio of prediction to deviation. ^d^ Bias = average deviations of cross-validation outputs from their reference values. ^e^ Slope = the gradient from plot of cross-validation outputs vs. their reference values. ^f^ Offset = the *y*-axis offset from plot of cross-validation outputs vs. their reference values.
